# Quiescence of human muscle stem cells is favored by culture on natural biopolymeric films

**DOI:** 10.1186/s13287-017-0556-8

**Published:** 2017-05-02

**Authors:** Claire Monge, Nicholas DiStasio, Thomas Rossi, Muriel Sébastien, Hiroshi Sakai, Benoit Kalman, Thomas Boudou, Shahragim Tajbakhsh, Isabelle Marty, Anne Bigot, Vincent Mouly, Catherine Picart

**Affiliations:** 1 CNRS, UMR 5628, LMGP, 3 parvis Louis Néel, F-38016 Grenoble, France; 2 Université de Grenoble Alpes, Grenoble Institute of Technology, 3 parvis Louis Néel, F-38016 Grenoble, France; 3 Université de Grenoble Alpes, Grenoble Institut des Neurosciences, GIN, Chemin Fortuné Ferrini, F-3800 Grenoble, France; 4 INSERM, U1216, F-38000 Grenoble, France; 5 Stem Cells & Development, Department of Developmental & Stem Cell Biology, Institut Pasteur, 25 rue du Dr Roux, Paris, 75015 France; 6 CNRS, UMR 3738; Institut Pasteur, 25 rue du Dr Roux, Paris, 75015 France; 7 Sorbonne Universités, UPMC Université Paris 06, INSERM UMRS974, CNRS FRE3617, Center for Research in Myology, 47 Boulevard de l’hôpital, 75013 Paris, France

**Keywords:** Quiescence, Satellite cells, Polymeric biomaterial, Layer by layer, Biomimetism, Culture platform

## Abstract

**Background:**

Satellite cells are quiescent resident muscle stem cells that present an important potential to regenerate damaged tissue. However, this potential is diminished once they are removed from their niche environment in vivo, prohibiting the long-term study and genetic investigation of these cells. This study therefore aimed to provide a novel biomaterial platform for the in-vitro culture of human satellite cells that maintains their stem-like quiescent state, an important step for cell therapeutic studies.

**Methods:**

Human muscle satellite cells were isolated from two donors and cultured on soft biopolymeric films of controlled stiffness. Cell adhesive phenotype, maintenance of satellite cell quiescence and capacity for gene manipulation were investigated using FACS, western blotting, fluorescence microscopy and electron microscopy.

**Results:**

About 85% of satellite cells cultured in vitro on soft biopolymer films for 3 days maintained expression of the quiescence marker *Pax7*, as compared with 60% on stiffer films and 50% on tissue culture plastic. The soft biopolymeric films allowed satellite cell culture for up to 6 days without renewing the media. These cells retained their stem-like properties, as evidenced by the expression of stem cell markers and reduced expression of differentiated markers. In addition, 95% of cells grown on these soft biopolymeric films were in the G0/G1 stage of the cell cycle, as opposed to those grown on plastic that became activated and began to proliferate and differentiate.

**Conclusions:**

Our study identifies a new biomaterial made of a biopolymer thin film for the maintenance of the quiescence state of muscle satellite cells. These cells could be activated at any point simply by replating them onto a plastic culture dish. Furthermore, these cells could be genetically manipulated by viral transduction, showing that this biomaterial may be further used for therapeutic strategies.

**Electronic supplementary material:**

The online version of this article (doi:10.1186/s13287-017-0556-8) contains supplementary material, which is available to authorized users.

## Background

In skeletal muscle, the tissue-specific stem cells—called satellite cells (SCs)—are normally quiescent but they can be recruited rapidly after an injury, providing the muscle with an ability to regenerate [1]. During injury, SCs are activated from the G0 phase (quiescence) and enter the cell cycle (G1 phase). Activated SCs that proliferate are called myoblasts, and the majority will fuse with the existing fiber or between themselves to regenerate the damaged tissue. However, not all SCs are committed to differentiation; some return to the G0 state to replenish the pool of quiescent SCs (self-renewal) [2–4]. Self-renewing SCs are characterized by expression of the specific marker *Pax7* and the absence of expression of myogenic differentiation markers such as myogenin (MyoG) [5].

Because of their high regeneration potential even for extended periods after the death of the patient [6], SCs have been used or envisaged for cell therapy for various dystrophies [7]. In some myopathies like Duchenne muscular dystrophy (DMD) where there is a deficiency in the structural protein dystrophin, a characteristic loss of SCs has been observed to be associated with repeated cycles of degeneration–regeneration, and allografts of SCs have been tested in DMD patients [8]. Although these early trials have given no overt clinical benefit to date, cell-based therapies still hold great promise in the treatment of muscular disorders. Current cell therapy involves several steps: muscle biopsy from a patient or a healthy donor and cell isolation, cell sorting using specific markers (e.g., CD56), in-vitro amplification in culture, possibly genetic correction and, finally, intramuscular reinjection of expanded cells in vivo [9]. Some convincing preclinical results have already been obtained for the treatment of oculopharyngeal muscular dystrophy by injection of autologous unmodified myoblasts [10, 11].

In spite of their therapeutic potential, the efficiency of SCs in cell therapy remains suboptimal. The ex-vivo culture of SCs, usually performed on plastic substrates, decreases their regenerative ability significantly because the SCs generate myoblasts [12]. Notably, the in-vitro culture of SCs results in their inevitable commitment to myoblasts and progressive transition from quiescent *Pax7* ^+^/MyoG^–^ cells to differentiated *Pax7* ^–^/MyoG^+^ cells [2]. The loss of *Pax7* during the in-vitro cell culture step is an indicator of the loss of the upstream stem-like state as SCs become preactivated myoblasts, thereby losing their cell therapeutic potential [9]. Unlike preactivated myoblasts, quiescent SCs exhibit robust regeneration capacity, a considerably higher engraftment rate as well as self-renewal potential following their transplantation in vivo [12, 13]. Thus, one major limiting step of current therapies for treatment of muscular myopathies is the phenotypic shift that occurs when SCs are cultured on typical plastic culture dishes [9, 12]. Cell therapy using SCs could be greatly improved by culturing donor cells ex vivo in appropriate culture conditions on biomaterials [13], mimicking in vitro the natural muscle environment, commonly called the “niche”, and thus maintaining a full regenerative potential. Consequently, clinical trials led to mild clinical improvements [7, 10].

Thus, from a therapeutic perspective, the prospect of preserving the regenerative potential of SCs ex vivo by avoiding activation would provide an innovative and efficient solution for the treatment of various forms of myopathies. There are few studies describing culture systems that are able to maintain the quiescent state of SCs [13–18]. In view of the difficulty in maintaining SC stem-like properties, the methods used to study cell quiescence do not generally involve cell culture, but rather isolation of cells by fluorescent (FACS) or magnetic (MACS) cell sorting devices [19], direct immunostaining of SCs on extracted fibers [14] or immunohistochemistry [15]. A concern is the relatively low number of cells that can be studied in these conditions, because SCs represent roughly 2–5% of the total adult muscle cells [16].

For these reasons, there is a need to define biomimetic culture substrates that would allow the study of SCs in the mid to long term. It is now widely recognized that the biophysical properties of the cellular environment dictate cell fate in vivo [17, 18] and in vitro [20–24]. A relevant culture environment mimicking the mechanical and biochemical properties of the SC niche may be key for maintaining stem cell properties ex vivo [12], either to study quiescence of human SCs or prior to their reinjection in a therapeutic context.

The current strategies used for the in-vitro study of muscle stem cells rely mostly on myoblast culture on surfaces coated with adhesive proteins or a mix of proteins such as Matrigel or gelatin [19, 25]. Adhesion proteins are widely used to mimic cell binding to the extracellular matrix (ECM), which is essential for muscle cell signaling [26]. Fibronectin expressed by SCs is known to modulate their expansion within their niche by potentiating Wnt7a signaling and transiently remodeling their environment [21]. In addition, the activation of specific cell receptors (e.g., integrins) by engineered material is a means to control cell adhesion, proliferation and differentiation [21, 27], and muscle stem cells were also shown to remodel their own ECM [28]. However, in addition to matrix composition, matrix stiffness (or rigidity) is also a determinant for muscle cell fate [29]. Indeed, a pioneering strategy using engineered multiwell platforms made of polyethylene glycol (PEG) with adapted rigidity was proposed [13]. At an optimal stiffness of 12 kPa, these PEG hydrogels were found to preserve 32% of SCs in their quiescent *Pax7* ^+^ state, in comparison with 6% for plastic wells [13]. This study opened new perspectives for the use of engineered matrices to control SC fate, including optimizing therapeutic strategies, by enabling the culture and amplification of SCs in a more physiological state. Recently, layer-by-layer films made of biopolymers poly(l-lysine) (PLL) and hyaluronan (HA) have shown promise in tissue engineering in view of their biomimetic properties [30] and established ability to control skeletal muscle cell differentiation [31, 32]. To control cell adhesion, the stiffness of these biopolymeric films can be modulated via tunable chemical crosslinking [33], which creates covalent peptide bounds between the two biopolymers. The mechano-chemical properties [33] and cell mechano-sensitivity of these films are well documented [31, 34, 35] and they allow cell culture over a long time period with no cytotoxicity [31].

In this work, we used these layer-by-layer films as biomaterial for SC culture. We cultured human SCs on (PLL/HA) biopolymeric films of controlled stiffness and studied their ability to remain in a quiescent state (G0) in vitro. This innovative approach allowed the maintenance of up to 85% of human SCs in a stem-like cell state characterized by *Pax7* expression, and up to 95% of cells in the G0/G1 state.

## Methods

### Reagents

PLL (26 kDa, P2636) was purchased from Sigma (St Quentin-Fallavier, France). HA of MW 360 kDa was purchased from Lifecore Biomedical (Chaska, MN, USA). For film crosslinking, 1-ethyl-3-(3-dimethylamino-propyl)carbodiimide (EDC) and *N*-hydrosulfosuccinimide (sulfo-NHS) were purchased from Sigma. Cell culture reagents were from Gibco (Gibco, Invitrogen, Cergy-Pontoise, France). Fetal bovine serum (FBS) was purchased from PAA Laboratories (Les Mureaux, France). Rhodamine phalloidin (P2141), polyclonal anti-fibronectin (F-3648) and monoclonal anti-skeletal myosin heavy chain (MHC; M4276) were purchased from Sigma. The antibody anti-*Pax7* was purchased from Developmental Studies Hybridoma Bank (DSHB) at the University of Iowa (Iowa City, IA, USA). Antibodies anti-myogenin (M225-sc576) and anti-cyclin D1 (H295, sc753) were purchased from Santa Cruz Biotechnology (Heidelberg, Germany). Anti-desmin antibody (clone D33) was purchased from Dako (Agilent Technologies, Les Ulis, France). Goat anti-type I Collagen-UNLB (1310-01) was purchased from SouthernBiotech (Clinisciences, Nanterre, France). Alexa Fluor 488-conjugated antibodies, Hoechst 33342 and the Prolong antifade Gold reagent were purchased from Molecular Probes-Invitrogen (Illkirch, France). The protease inhibitor cocktail (Complete mini) was purchased from Roche (Boulogne-Billancourt, France). Horseradish peroxidase-conjugated anti-mouse (NA931) and anti-rabbit (NA934) secondary antibody were purchased from GE Healthcare (Vélizy-Villacoublay, France).

### Film preparation and crosslinking procedure

The films were prepared as described previously [33] with an automated dipping machine (Dipping Robot DR3; Kirstein GmbH, Germany) on 14-mm diameter (for optical and electron microscopies) and 32-mm diameter (for western blot analyses) glass slides (VWR Scientific, France). PLL at 0.5 mg/ml, HA at 1 mg/ml and polyethyleneimine (PEI; precursor layer) at 2 mg/ml were dissolved in Hepes–NaCl buffer (20 mM Hepes at pH 7.4 and 0.15 M NaCl). During the film formation, all of the rinsing steps were performed with a rinsing solution (0.15 M NaCl, pH 6.5). Twelve pairs of layers were deposited on the glass slides, thus forming a (PLL/HA)_12_ film. After build up, films were crosslinked following a protocol published previously [33] using EDC at various concentrations (5, 10, 50 and 70 mg/ml) and sulfo-NHS (kept constant at 11 mg/ml). The crosslinking level of the films then referred to EDC concentrations: EDC*X* indicates a film crosslinked with EDC at *X* mg/ml. The films were incubated overnight at 4 °C in the crosslinking solution (0.15 M NaCl at pH ~ 5.5). Finally, the films were rinsed three times with Hepes–NaCl buffer.

### Cell culture

Human muscle biopsies were obtained from the bank of tissues for research Myobank-AFM (code BB-0033-00012), a partner in the EU network EuroBioBank, in accordance with European recommendations and French legislation. Primary human muscle SCs were isolated from quadriceps biopsies of two male donors: a 53-year-old individual and a 55-year-old individual. Human myoblast cultures were prepared by finely mincing the biopsies and plating the resulting explants in a drop of FBS, as described previously [36]. Cells migrating out of the explants were then expanded and the remaining explants were discarded. The purity of the culture was monitored using desmin expression detected by immunofluorescence, or by FACS using anti-CD56 antibody. The myogenicity was 73% for the 53-year-old individual and 80% for the 55-year-old individual.

The cells were cultured on polymeric films from division 12. For the FACS experiments, the cells used were a clone of human myoblasts from the 53-year-old donor, immortalized as already described [37].

The primary and immortalized cells were cultured in growth medium (GM) containing DMEM:Medium 199 (4:1) supplemented by 25 μg/ml fetuin, 5 μg/ml insulin, 0.2 μg/ml dexamethasone, 0.5 ng/ml basic fibroblast growth factor (FGF), 5 ng/ml human epidermal growth factor (EGF), 20% FBS and 1% penicillin/streptomycin. For differentiation experiments, cells were seeded on films and on tissue culture polystyrene (called plastic) and cultured for 3 days in GM to reach 80% confluence. The medium was then switched to differentiation medium (DM) containing DMEM with 1% penicillin/streptomycin. For all experiments, cells were plated at a density of 150,000 cells/cm^2^.

### Scanning electron microscopy

Cell morphology on polyelectrolyte films or plastic was imaged using scanning electron microscopy with an FEI-QUANTA250-SEM-FEG and an Everhart Thornley Detector (ETD). Cells were washed in PBS and fixed with 3.3% glutaraldehyde in cacodylate buffer (0.1 M cacodylate–HCl, pH 7.2) for 1 h at room temperature and then washed once using the same buffer. The samples were dehydrated using a graded ethanol series from 30 to 100%, and were finally air-dried overnight at 37 °C. A carbon coating was deposited onto the samples prior to imaging (Denton Vacuum DESK IV). The images were acquired under high-vacuum mode at an acceleration voltage of 5 kV.

### Immunofluorescence

For staining of *Pax7*, myogenin and skeletal MHC, cells were fixed with 3.7% formaldehyde in PBS for 20 min and permeabilized for 4 min in 0.2% Triton X-100 in TBS (0.15 M NaCl, 50 mM Tris–HCl, pH 7.4). Samples were blocked in TBS containing 0.1% BSA for 1 h, and were then incubated with rabbit anti-myogenin (1:100), mouse anti-*Pax7* (1:10), mouse anti-MHC (1:200), rabbit anti-fibronectin (1:100) or goat anti-collagen I (1:40) antibodies in TBS with 0.2% gelatin for 30 min. AlexaFluor488-conjugated secondary antibody (1:200) was then incubated for 30 min. For actin staining, cells were incubated for 30 min with phalloidin-rhodamine (1:800). Nuclei were stained with 5 μg/ml Hoechst 33342. All of the samples were mounted onto coverslips with Prolong antifade reagent and viewed using a fluorescence microscope (Axiovert 200 M; Zeiss, Germany) equipped with a Coolsnap EZ CCD camera and Metaview software (both from Ropper Scientific, Evry, France) or using a confocal Zeiss LSM 700 microscope.

### Quantification of extracellular matrix proteins

Collagen and fibronectin were immunostained for quantification. Analysis of images was performed using ImageJ 1.44p (NIH, Bethesda, MD, USA). In each experiment, at least 100 cells were analyzed per condition. Actin staining was always used as a reference to define the cell spreading area, which was used as a mask to obtained binary images. The whole cell area was black (pixel intensity = 1) and the background outside the cells was white (pixel intensity = 0). The masks were then applied on collagen and fibronectin immunostaining images in order to quantify the expression of ECM proteins by the cells. The total integrated fluorescence density for a given image was normalized to the total cell number. The background noise was always set to zero.

### Viability and proliferation assays

Viability assay was performed by exclusion of vital dyes using the Live/Dead Viability/Cytotoxicity assay (L3224; Life Technologies). Calcein AM (2 μM) and 4 μM ethidium homodimer-1 (EthD-1) were added directly to cells and incubated for 10 min at 37 °C in GM before taking the pictures using the 20× objective of the fluorescence microscope. Proliferation was assessed by a 5-ethynyl-2′-deoxyuridine (EdU) proliferation assay (Click-iT® EdU Alexa Fluor® 647 Imaging Kit, C10340; Life Technologies) according to the manufacturer’s instructions. The SCs were incubated for 24 h with 10 μM EdU prior to fixation.

### Cell cycle analysis by FACS

Human muscle cells were fixed in 70% EtOH. The cells were then washed twice by centrifugation at 700 rpm at 4 °C before suspension in PBS containing 50 μg/ml propidium iodide (PI) and 0.2 mg/ml RNAse [38]. The samples were analyzed with a BD Accuri C6 (Becton Dickinson Biosciences, France) and the plots were optimized with the cell cycle analysis tool of FCS Express 4 software (De Novo Software, Glendale, CA, USA).

### Polyacrylamide gel electrophoresis and immunoblotting

Primary muscle cells were seeded at 20,000 cells/cm^2^ in six-well plates with or without the biopolymeric films, and were allowed to grow for 1 or 3 days on plastic and EDC70 films and for 1, 3, 6 or 10 days on EDC10 films. At each time point, cells were scraped, rinsed in PBS and then lysed in the lysis buffer (50 mM Tris at pH 8.0, 150 mM NaCl, 1.0% Triton X-100 and 1× protease inhibitor cocktail). After boiling, protein samples were resolved on 12% polyacrylamide gels before transfer onto PVDF membranes (GE Healthcare, UK). Membranes were then saturated in 5% milk in TBS that contained 0.1% Tween 20 for 1 h and subsequently incubated with monoclonal antibodies against *Pax7* (1:100), myogenin (1:500), cyclin D1 (1:200) and β-tubulin (1:1000). Membranes were washed and incubated with a horseradish peroxidase-conjugated anti-mouse secondary antibody (1:20,000). Peroxidase activity was visualized by autoradiography.

### Viral transduction

Lentivirus GFP was produced in HEK293T cells transfected with psPAX2 (addgene #12260), pCMV-VSVG (addgene #8454) and pWPXLD (containing GFP, addgene #12258) vectors. Primary human muscle cells were seeded on EDC10 films or plastic, and lentivirus transductions were performed 24 h later. The multiplicity of infection (MOI = number of viral particles/cell) was 120. Twenty-four hours after viral transduction, the cells were replated on plastic, and pictures were acquired before and after replating with the same settings.

### Data representation and statistical analysis

All data are expressed as mean ± SEM, except for box plots. For box plots, the edges of the box represent the 25th and 75th quartiles and the whiskers represent the 10th and 90th centiles. The data were compared using one-way analysis of variance (ANOVA). Differences were considered significant when *p* < 0.05, and highly signficant from *p* < 0.01.

## Results

### Biopolymeric film stiffness modulates the morphology of primary human muscle cells

**Fig. 1:**
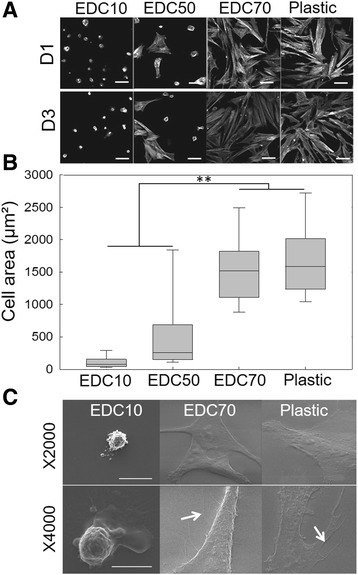
SC morphology on biopolymeric films in comparison with plastic. Biopolymeric films were crosslinked at different levels: EDC10, EDC50 and EDC70. **a** Representative images of actin staining after 1 day (D1) or 3 days of culture (*D3*) on EDC10, EDC50 and EDC70 films and plastic. *Scale bars*: 50 μm. **b** Corresponding quantification of the cell spreading area. Data plotted from three independent experiments (*n* = 200 cells minimum per experimental condition). **p < 0.01. **c** Scanning electron microscopy imaging of SCs cultured on EDC10 films, EDC70 films and plastic. The white arrows indicate filopodia. *Scale bars*: 30 μm

Cell adhesion and spreading was first assessed for SCs cultured onto the biopolymeric (PLL/HA)_12_ films with varying crosslinking levels from EDC10 to EDC70 and compared with plastic as the control condition. Adapting the crosslinker concentration enabled us to modulate film stiffness as described previously [37] because the crosslinking level is linked to the film stiffness. The elastic modulus of the (PLL/HA)_12_ films ranges from ~30 kPa for EDC5 to ~400 kPa for EDC70 as described previously [33]. SCs exhibited different morphologies depending on the film stiffness (Fig. 1a). On EDC5 films, SCs adhered poorly and were washed away during the fluorescent staining procedure (data not shown). On EDC10 films, SCs were round with no protrusion or actin stress fibers. On the mild crosslinked films (EDC50), there was a mix of round as well as spread cells. In contrast, cells on EDC70 films were highly spread with numerous actin stress fibers (Fig. 1a). Notably, the cell area on EDC70 films was similar to that on plastic, but was ~20 times higher than on EDC10 films and ~6 times higher than on EDC50 films (Fig. 1b). Imaging of individual cells by scanning electron microscopy revealed distinct phenotypes (Fig. 1c): SCs were blebbing on EDC10 films while they formed lamellipodia and filopodia on stiffer EDC70 films and plastic. Thus, SCs are sensitive and showed phenotypic variations according to biopolymeric film stiffness.

We next investigated whether round cells on the EDC10 films were viable for extended periods. To this end, SCs were cultured on EDC10 films over 21 days in GM without medium replenishment. Calcein staining showed that SCs were viable, but we observed that SCs spread after 10 days in these conditions (Additional file 1: Figure S1). Based on these results, we selected the two extreme conditions of low cell spreading (EDC10 films) and high cell spreading (EDC70 films) for the rest of the study.

### Fibronectin secretion is related to cell spreading

**Fig. 2:**
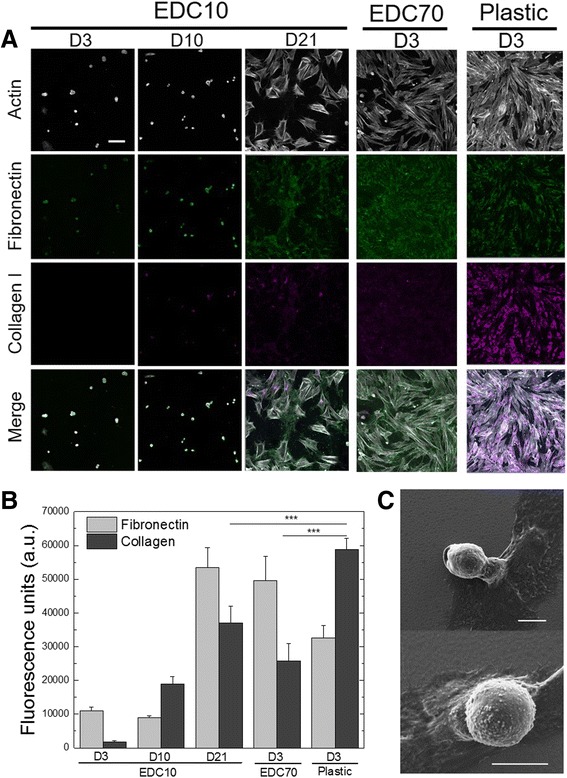
SC morphology and ECM protein secretion on biopolymeric films. EDC10 and EDC70 films were compared with plastic. **a** Representative pictures of actin, fibronectin and collagen I staining after culture for 3, 10 and 21 days (respectively *D3*, *D10* and *D21*) on EDC10 films, and for 3 days on EDC70 films and plastic. *Scale bar*: 50 μm. **b** Quantification of fibronectin and collagen I secretion by SCs cultured on EDC10 films (D3, D10, D21), EDC70 films and plastic (D3). Data are mean ± SD from two independent experiments (*n* = 100 cells minimum per condition). ***p <0.001. **c** Scanning electron microscopy micrographs of SCs seeded on EDC10 films for 10 days. *Scale bars*: 30 μm

To further investigate the slow spreading phenotype of SCs on EDC films, we quantified the secretion of two principal proteins of the ECM: fibronectin and collagen I (Fig. 2). Fibronectin is an important component of the SC niche and its secretion by SCs may activate the cells by remodeling the niche [21]. Collagen I is an important component of the muscle fiber and contributes to stabilize the muscle tissue [39]. Collagen I is also known to be a major component of the fibrotic tissue influencing cell differentiation and is secreted by mouse SCs in their niche, as well as by other cell types [40]. Figure 2a shows SC spreading and secretion of fibronectin and collagen I after 3, 10 and 21 days in culture in GM, as revealed by immunostaining. At day 3, SCs cultured on plastic secreted 2 and 50 times more collagen I than when they are cultured on EDC70 and EDC10 films, respectively. Higher resolution imaging confirmed that collagen I is expressed at higher levels by cells cultured on plastic compared with films (Additional file 1: Figure S2). In contrast, SCs secreted ~30% more fibronectin when cultured for 3 days on EDC70 films and for 21 days on EDC10 films, in comparison with plastic (Fig. 2b). On EDC10 films, there was a sharp increase in fibronectin secretion between days 10 and 21, where the surface was covered by fibronectin. Analysis by scanning electron microscopy showed that SCs remained round and adherent until they were spread on EDC10 films after 10 days (Fig. 2c).

### *Pax7* expression is sustained on EDC10 films while MyoG remains low

*Pax7* expression has been demonstrated in quiescent and proliferating SCs; it is then downregulated during myogenic commitment and differentiation as MyoG expression increases [5, 41]. These markers enable the classification of myogenic cells as *Pax7* ^+^/MyoG^–^ and committed myoblasts as *Pax7* ^–^/MyoG^+^ [12, 42].

**Fig. 3:**
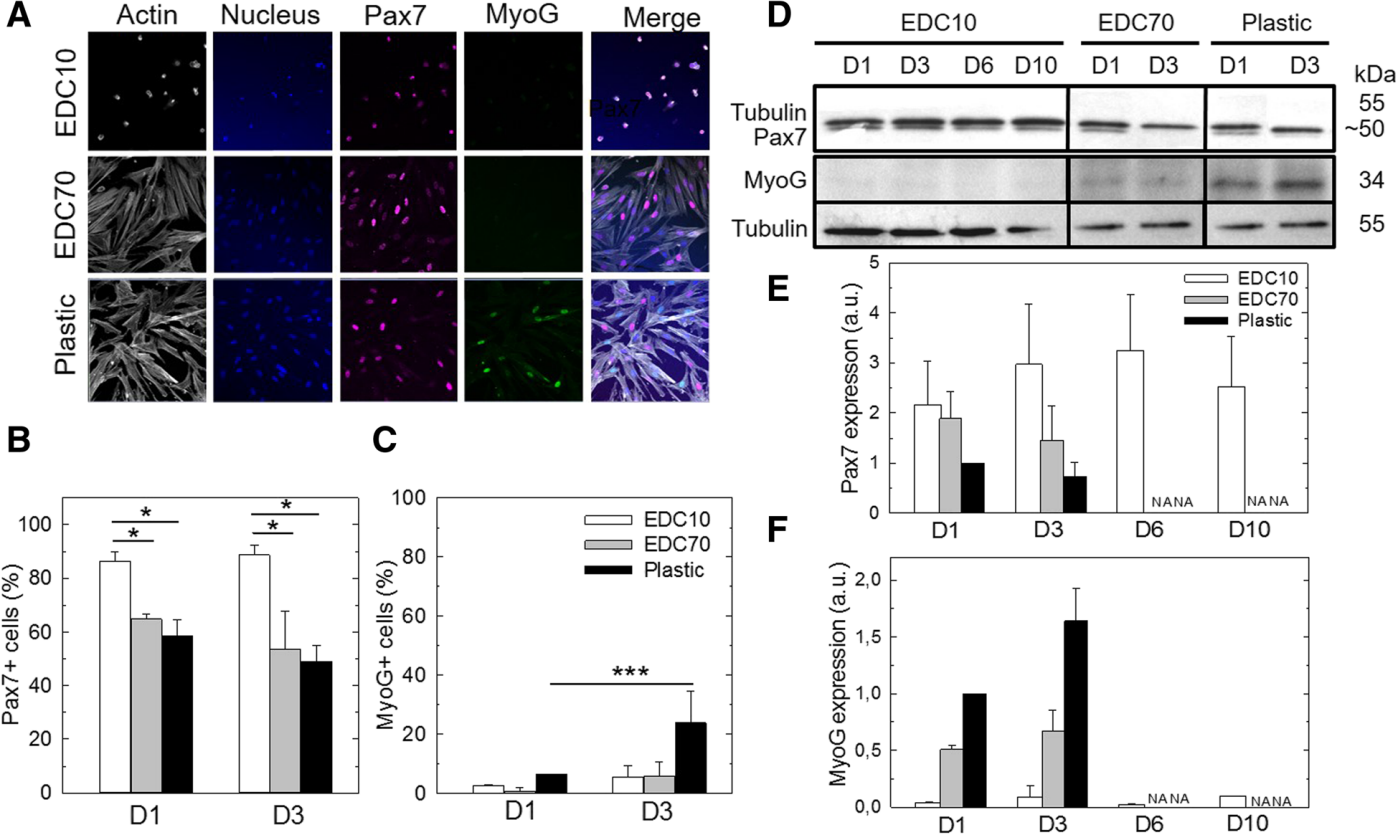
*Pax7* and myogenin expression in SCs cultured on biopolymeric films. **a** Fluorescent labeling of actin, *Pax7*, myogenin (*MyoG*) and nucleus (Hoechst) at D3 on EDC10 and EDC70 films in comparison with plastic. *Scale bar*: 50 μm. **b** *Pax7* and **c** MyoG expression were quantified by immunofluorescence at D1 and D3 in GM. Data from three independent experiments (*n* = 100 cells minimum per experimental condition). *p < 0.05, ***p < 0.001. **d** Western blot analysis of Pax7 and MyoG expression and semi-quantitative determination of **e** *Pax7* and **f** MyoG expression as compared with plastic control (the condition “plastic D1” was set to 1). Results of D6 and D10 are only given for the culture on EDC10 films because cell confluence was reached on EDC70 films and plastic as soon as day 3; for these conditions, D6 and D10 are thus referred to as “not applicable” (*NA*). Data are mean ± SD from three independent experiments. *D1*, *D3*, *D6*, *D10* days 1, 3, 6, 10

The myogenic status of SCs cultured on biopolymeric films and plastic was determined by immunofluorescence and western blotting (Fig. 3). Figure 3a shows immunofluorescence of *Pax7* and MyoG after 3 days of culture on EDC10 and EDC70 films and plastic. The proportion of *Pax7*-expressing SCs was maintained at 88 ± 5% after 3 days in culture on EDC10 films while it significantly decreased to 54 ± 14% on EDC70 films and 49 ± 6% on plastic (*p* < 0.005) (Fig. 3b). Moreover, the proportion of cells expressing MyoG after 3 days was negligible on both films compared with the expression obtained on plastic (~5 ± 4% on films and 24 ± 10% on plastic) (Fig. 3c). These results indicate that SCs cultured on EDC10 films do not differentiate and are maintained in a stem-like (*Pax7* ^+^/MyoG^–^) or quiescent state. The expression of *Pax7* and MyoG was confirmed by western blot analysis. On plastic, SCs lost *Pax7* expression rapidly and, concomitantly, strongly expressed MyoG (Fig. 3d, e). On EDC70 films, *Pax7* decreased between days 1 and 3, while MyoG expression increased. In contrast, *Pax7* expression was sustained over 10 days of culture on EDC10 films while MyoG expression was negligible, compared with EDC70 films and plastic (Fig. 3d, e). Taken together, these data suggest that SCs maintain a stem-like state when cultured for 10 days on EDC10 films.

### A nonproliferative state is maintained for 10 days on EDC10 films

**Fig. 4:**
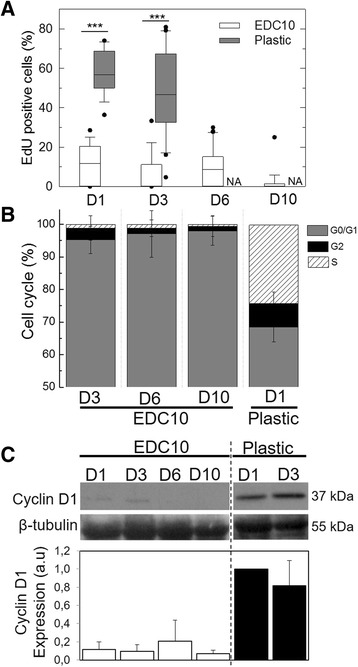
Absence of SC proliferation on EDC10 films up to 10 days. **a** Quantification of SC proliferation by EdU assay: for D1, D3, D6 and D10 on EDC10 films and only for D1 and D3 on plastic, because confluence was reached (referred as “not applicable” (*NA*)). Data are box plots from three independent experiments (*n* = 200 cells in total per experimental condition). ***p < 0.001. **b** FACS analysis of the cell cycle at D3, D6 and D10 on EDC10 films and at D1 on plastic. Data plotted from two independent experiments (*n* = 5000 cells per condition). **c** Western blot of cyclin D1 and semi-quantitative analysis. The condition “plastic D1” was taken as the reference (value set to 1). Data are mean ± SD from three independent experiments. *D1*, *D3*, *D6*, *D10* days 1, 3, 6, 10, *EdU* 5-ethynyl-2′-deoxyuridine

To further characterize the status of SCs cultured on EDC10 films, the proliferation state and cell cycle were analyzed after 1, 3, 6 and 10 days of culture (Fig. 4). The proliferation, assessed by EdU incorporation, was maintained below 10% on EDC10 films while 58 ± 5% of SCs on plastic retained the label at day 1 (Fig. 4a). The proportion of cells confined to the G0/G1, G2/M or stationary S phase of the cell cycle was determined by flow cytometry after DNA labeling with propidium iodide (Fig. 4b). The proportion of cells confined in the G0/G1 phase was elevated on EDC10 films (>95%) and remained so until 10 days, in comparison with plastic where only 68 ± 4% remained in G0/G1 at day 1. In contrast, the S phase was hardly detectable in SCs cultured for 3 days on EDC10 films, while 24% of SCs cultured on plastic were in the S phase, as shown previously for C2C12 myoblasts [43]. Similarly, the proportion of SCs in the G2/M phase decreased for EDC10 films and was negligible after day 6, while 7 ± 3% of SCs cultured on plastic were in the G2 phase.

To discriminate the quiescent G0 state from the early activated G1, the expression of cyclin D1, a protein promoting the entry into the cell cycle [44], was determined by western blot analysis (Fig. 4c). Cyclin D1 regulates the initiation of cell cycle progression in quiescent cells and is expressed in the G1 phase to initiate DNA synthesis [44]. From day 1 to day 10, cyclin D1 was barely detected in SCs cultured on EDC10 films, while it was highly expressed by SCs cultured on plastic as early as day 1. Taken together, these results suggest that most of the SCs cultured on EDC10 films are not G1 state but most likely in a G0 phase.

### In-vitro activation and differentiation of quiescent SCs

**Fig. 5:**
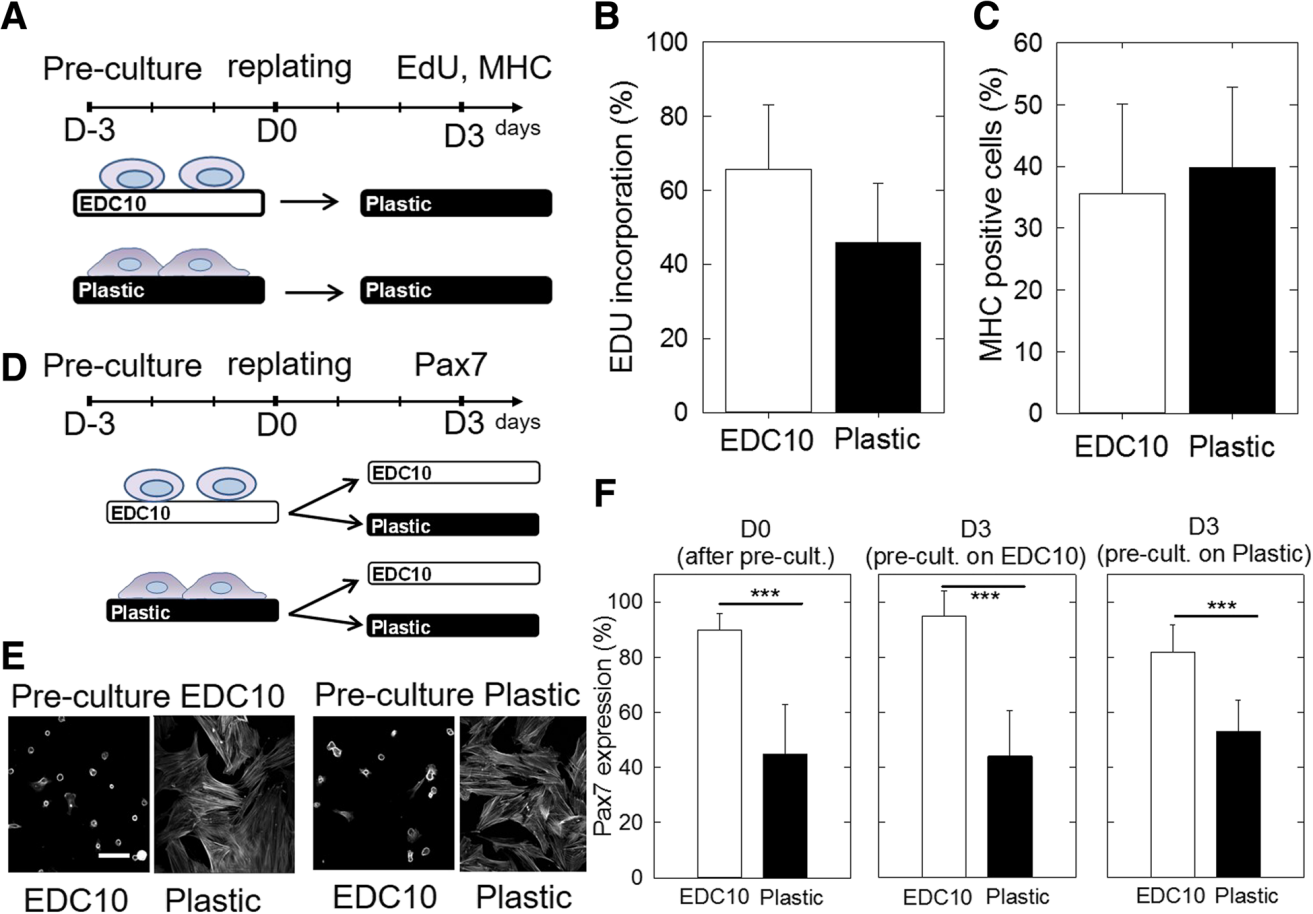
Reactivation of cells cultured on EDC10 films. **a** Schematic of experiment where SCs were precultured on EDC10 films or plastic for 3 days and then replated onto plastic. **b** Proliferation was assessed by EdU incorporation and **c** differentiation by the number of MHC^+^ cells after 3 days in DM. Data are mean ± SD from two different experiments (*n* = 50 cells per condition in each experiment). **d** Schematic of experiment where SCs were precultured either on EDC10 films or plastic for 3 days. SCs were then replated on EDC10 films and plastic for another 3 days. The cells were fixed and stained for actin and *Pax7*. **e** Representative images of actin staining. *Scale ﻿bar*﻿: ﻿50 ﻿μm﻿. **f** Number of *Pax7*-positive cells: after the preculture (at D0), on EDC10 films and plastic, after replating for 3 days and depending on the preculture conditions (EDC10 films or plastic). Data are mean ± SD from three independent experiments (*n* = 100 cells). ***p < 0.001. *D–3*, *D0*, *D3*, days –3, 0, 3, *EdU* 5-ethynyl-2′-deoxyuridine, *MHC* skeletal myosin heavy chain

To determine whether these cells could reenter the cell cycle after culture on EDC, nonproliferative SCs precultured on EDC10 films were replated onto plastic to trigger cell activation. Their reentry in the cell cycle was assessed by EdU incorporation and their differentiation to myocytes by expression of the myogenic marker myosin heavy chain (MHC). Cell reactivation was evaluated by replating SCs from EDC10 films to plastic and was compared with those cells replated from plastic to plastic as control (Fig. 5a). Three days after replating on plastic, SCs started to proliferate independently of the substrate used for their preculture (EDC10 films or plastic) (Fig. 5b): 65 ± 17% of SCs precultured on EDC10 films and 45 ± 16% of those precultured on plastic incorporated EdU, with no significant difference between the two conditions. In addition, after 3 days of culture in differentiation medium, 30–40% of SCs expressed MHC—similarly to if they had been precultured on EDC10 films (35 ± 14%) or on plastic (40 ± 13%) (Fig. 5c).

The reversibility of *Pax7* expression before and after replating was also assessed by immunofluorescence (Fig. 5d–f and Additional file 1: Figure S3). The cells were precultured for 3 days on EDC10 films or plastic (as control), before being detached and replated on other EDC10 films or on plastic (Fig. 5d). Cells spread solely when they were replated on plastic (Fig. 5e and Additional file 1: Figure S3), independent of the initial preculture material (EDC10 films or plastic). Conversely, cells on EDC10 films remained rounded in shape, even if they had been precultured on plastic (Fig. 5e and Additional file 1: Figure S3). In addition, the number of *Pax7* ^+^ cells after replating depended solely on the material substrate onto which SCs were cultured for the last 3 days, and not on the material substrate used for the preculture step (Fig. 5e and Additional file 1: Figure S3). Indeed, more than 80% of SCs were *Pax7* ^+^ on EDC10 films (95 ± 9% after preculture on EDC10 films and 82 ± 10% after preculture on plastic) while only ~45–55% were *Pax7* ^+^ on plastic (45 ± 18% after preculture on EDC10 films and 53 ± 11% after preculture on plastic). This result highlights two critical points: first, there was a correlation between the frequency of *Pax7* ^+^ cells and the nature of the underlying substrate, cells cultured on EDC10 films being a more favorable state for *Pax7* ^+^ expression compared with plastic (45–55% of *Pax7* ^+^ cells after 3 days of culture); and, second, *Pax7* expression was reversible in SCs, at least within the first 3 days of culture. In other words, cells precultured on plastic can recover their *Pax7* expression if they are cultured on EDC10 films.

Therefore, for SCs precultured for 3 days on a given material substrate, there was no permanent memory of that material; cellular plasticity was maintained, however, allowing the recovery of a naïve *Pax7* ^+^ state, provided SCs are cultured on the *Pax7* ^+^-enabling EDC10 films. This recovery may occur by upregulation of *Pax7* expression, or by selection of cells that were still *Pax7* ^+^ cells on plastic and were predisposed to survive on EDC10 films.

Taken together, these results show that SCs precultured on EDC10 films can reactivate, proliferate and differentiate as efficiently as when precultured on plastic, indicating that EDC10 films promote cellular quiescence. Furthermore, in our experimental settings, the quiescent or proliferative state of SCs can be reversed: quiescent cells can become proliferative and, inversely, proliferative cells can return to quiescence, demonstrating the plasticity of SCs in vitro.

### Quiescent cells can be transduced by lentivirus

**Fig. 6:**
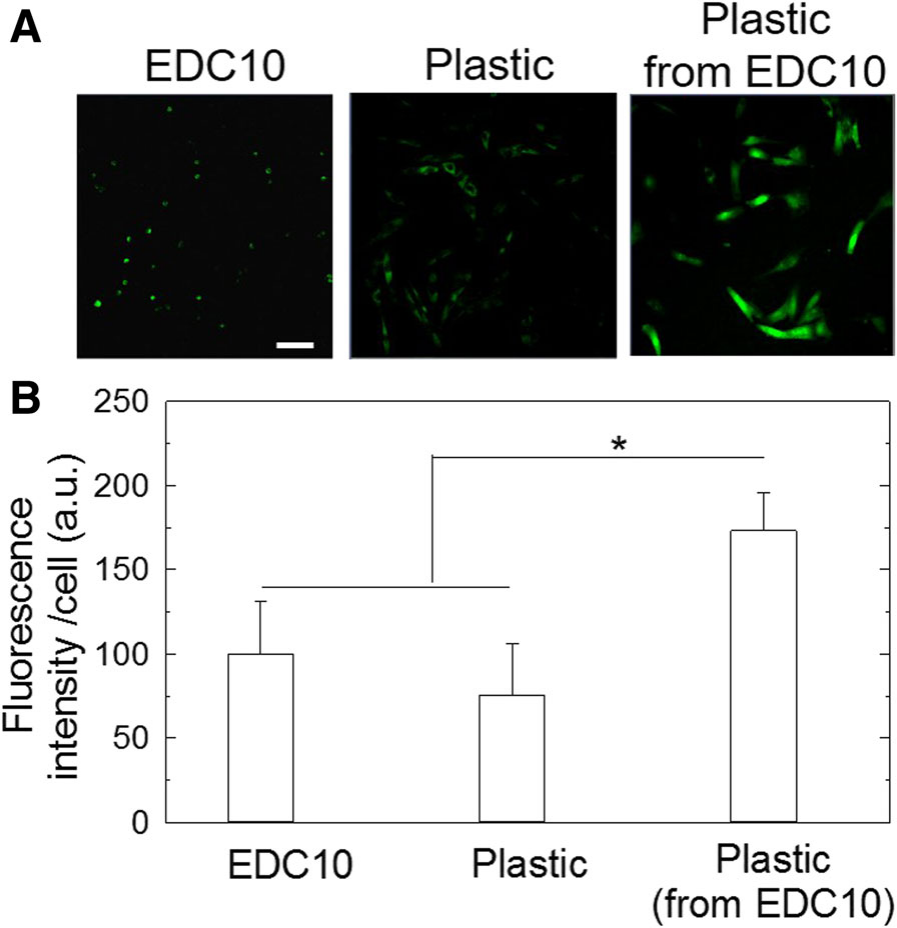
Transduction of SCs by lentivirus. **a** Confocal images of SC-transduced lentivirus GFP (MOI = 120) after 1 day of culture on EDC10 films or plastic. *Scale bar*: 100 μm. **b** Quantification of the fluorescence intensity on EDC10 films or plastic, measured 24 h after transduction and 24 h after replating on plastic (following a preculture on EDC10 films). *p < 0.05

Finally, to show the potential of quiescent cells for gene therapy studies, we performed a proof-of-concept experiment and transduced SCs with a viral vector (Fig. 6). To this end, quiescent SCs cultured either on EDC10 films or on plastic, or cultured on plastic after a preculture on EDC10 films, were transduced with lentivirus encoding GFP (Fig. 6a). The lentivirus efficiently transduced the quiescent SCs after 24 h. Notably, GFP expression was significantly higher (by 1.8-fold) when SCs had been precultured on EDC10 films prior to being cultured on plastic, in comparison with SCs cultured only on EDC10 films (set arbitrarily to 1) or plastic (0.75 times lower than the normalized condition) (Fig. 6b). These results show that SCs can be transduced by lentivirus and that the efficacy of transfection is maximal when cells are precultured on EDC10 films.

## Discussion

In resting muscle, SCs are arrested in a reversible quiescent phase and they have capacity to rapidly reactivate in response to injury [45]. The molecular mechanisms involved in G0 exit have recently been the subject of intense studies [15, 46–48]. However, SCs are difficult to maintain in this quiescent state in vitro and the molecular analysis of quiescent SCs on the materials used as culture substrates (tissue culture polystyrene, plastic coated with proteins) is technically challenging due to the spontaneous shift toward commitment. Thus, most of the recent studies of quiescence in SCs are performed either directly in vivo [15, 46], in situ with SCs on the fiber [47] or ex vivo but a few hours after isolation [49], already with a potential and variable shift toward an activated state. These conditions greatly limit the amount of available cells and the full characterization of the studied cells. Furthermore, it is difficult to make direct comparisons between these studies due to the unknown phenotypes of SCs under these various conditions. A key to control SC behavior may be to modulate the biophysical properties of the in-vitro microenvironment because these are known to influence cell fate [20].

Using a biopolymeric platform for the in-vitro culture and study of adherent quiescent SCs, we show that human SCs are sensitive to the crosslinking level of the biopolymeric films, as reported previously for mouse skeletal myoblasts [31]. While SCs in culture spread on plastic or stiff substrates, they remain round and exhibit blebs on EDC10 films. These round-blebbing cells are reminiscent of the SC shape reported previously in vivo during migration [50]. This mechanism is indeed poorly studied ex vivo due to the high SC cell spreading on plastic culture substrates and due to the difficulty of imaging SCs on a single fiber at high resolution. Our results suggest that SCs cultured ex vivo on soft EDC10 biopolymeric film maintained a phenotype close to their native state in vivo via physical signals.

The maintenance of the quiescent state from day 1 to day 10 for SCs cultured on EDC10 films was shown by several concomitant findings: sustained *Pax7* expression without expression of MyoG; absence of proliferation; confinement of more than 95% of SCs in the G1/G0 phase; the absence of cyclin D1 expression, which suggests that the cells are in the G0 phase; and cell reactivation after replating on plastic. Previous studies showed that SCs can also enter a deeper state of quiescence, called dormancy [6]. Here, we showed the possibility to reactivate SCs by replating them on plastic, exploiting the reversible process of exiting and entering the cell cycle. Importantly, we showed that the proportion of quiescent cells in the population increases significantly after preculture on plastic and replating on EDC10 films, either by upregulation of *Pax7* or selection of a preexisting *Pax7* ^+^ population. These results also indicate that amplification on plastic is possible prior to culture on EDC10 films to recover a larger population of quiescent cells.

Interestingly, short-term culture of SCs on EDC10 films resulted in round morphology associated with low fibronectin and collagen I secretion, while SCs cultured on plastic secreted significantly more collagen I. However, longer-term culture on EDC10 films resulted in some cell spreading, which was associated with the secretion of ECM proteins. We hypothesize that fibronectin secreted by SCs in GM hides the mechanical cues, allowing the cells to spread independently of the underlying substrate. It is likely that the biochemical signal of the fibronectin layer overrides the mechanical signal from EDC10 films. This result sheds some light on the role of the underlying material substrate in the secretion of ECM proteins by SCs: both the mechanical and the biochemical microenvironment of SCs can influence their morphology and protein secretion, as well as their fate. Fibronectin expressed by SCs is known to modulate their expansion within their niche by potentiating Wnt7a signaling and transiently remodeling their environment [21]. In addition, the activation of specific cell receptors (e.g., integrins) by engineered materials is a means to control cell adhesion, proliferation and differentiation [21, 27], and muscle stem cells were also shown to remodel their own ECM [28].

Mechanical signals have been shown to be as important as biomechanical signals [51]. Mechanical properties are often characterized by the stiffness (also called rigidity) of a material, which has been shown to be a crucial parameter in stem cell fate determination [20] and SC self-renewal [13]. Plastic traditionally used for cell culture is a very stiff material (~3 GPa). In contrast, the elasticity of muscle was found to be ~12 kPa [29], the elasticity of undifferentiated C2C12 myoblasts ~11 kPa and the elasticity of isolated myofibers 45 kPa [52]. Thus, there are approximately five orders of magnitude of difference between muscle stiffness and the traditional cell culture substrate.

Our results are in agreement with a previous study showing that there was an optimal stiffness of PEG hydrogels (~12 kPa) to foster SC self-renewal in culture and sustained *Pax7* expression [13]. These PEG hydrogel substrates allowed the maintenance of *Pax7* expression in 32% of the daughter cells, which is below the frequency reached on soft biopolymeric films.

Recently, Schroder and coworkers proposed a new culture model by culturing SCs in a semi-solid suspension medium of methyl cellulose [53]. Interestingly, the authors suggested that the absence of cell adhesion to the material substrate triggered the cells to enter G0 arrest. This is reminiscent of our findings of a low adhesive/round state of SCs cultured on EDC10 films. Because their study was done over 4 days [53], it would be interesting to further evaluate the effective capacity of SCs to remain quiescent over longer periods of time. Although very interesting, this culture model does not provide morphological information for SCs, because the cells need to be cytospun for microscopic observation [53]. Another study used a cocktail of molecules (drugs, proteins, growth factors and other biomolecules) combined with an engineered 3D microscaffold to mimic a muscle fiber [48]. In this artificial niche with a well-defined cell culture medium, cells can remain quiescent. However, the effects of all the biochemical signals composing the cocktail of bioactive molecules would need to be tightly controlled in the long term.

These strategies provide information on the native state of SCs but none of them featured an adherent culture platform, such as the biopolymeric films, for the ex-vivo culture of quiescent SCs to facilitate translation studies. According to Negroni et al. [7], the ideal cell candidate for cell therapy should fit the following requirements: high number, easy isolation and amplification, high myogenic potential, specific homing capacities [54] and genetically modifiable. The high regeneration potential demonstrated by quiescent SCs in their niche implanted in vivo implies that, for ex-vivo culture and retransplantation, a high amplification rate is not needed to obtain billions of injected cells. In view of the regenerative potential of SCs, as few as seven SCs associated with one transplanted myofiber can generate over 100 new myofibers, because these cells are able to expand in vivo and self-renew relatively efficiently [54]. Thus, quiescent SCs are among the most promising candidates for muscle cell therapy [55].

SCs cultured on EDC10 appear to fulfill several requirements for cell therapy because they are quiescent, can be preamplified by culture on plastic and are transduced by lentivirus. Notably, in our experimental conditions, the precultured cells on EDC10 films exhibited the highest transduction capacity. The biopolymeric EDC10 film may thus be further explored in the context of cell therapy: the autologous patient cells may be first preamplified on plastic, possibly reprogrammed genetically, before being replated on EDC10 films to help them recover their quiescent state, prior to transplantation. Alternately, transduction may be done after replating on EDC10 films and prior to transplantation.

## Conclusion

We present an innovative biomimetic material for the maintenance of the quiescent state of human muscle stem cells. This was shown by sustained expression of the stemness marker *Pax7* and an absence of entry in the cell cycle. Furthermore, after short-term preculture on plastic that tends to compromise regenerative potential, muscle stem cells cultured on the soft biopolymeric film could recover their *Pax7* expression. Muscle stem cells grown on the soft biopolymeric films could also be transduced efficiently by a lentivirus. Taken together, these results open perspectives for use of these soft biopolymeric films in future cell therapies to repair damaged muscles.

## Additional file

Additional file 1:
**Figure S1.** SCs are alive after 21 days of culture. Calcein/EthD (Live/dead) assay on SCs during 21 days on EDC10 films without any change/refreshing ﻿of ﻿GM. *Scale bar*: 100 µm. **Figure S2.** SCs secrete more Collagen I on plastic than on EDC10 films. Immunostaining of fibronectin and collagen I in SCs cultured 21 days on EDC10 films, 3 days on EDC70 fil﻿ms and plastic. Actin was stained by rhodamine-phalloidin. *Scale bar*: 200 µm. **Figure S3.** Images of the cells corresponding to Figure 5, for DAPI and Pax7 staining.

## Abbreviations

DM: Differentiation medium
DSHB: Developmental Studies Hybridoma Bank
ECM: Extracellular matrix
EDC: 1-Ethyl-3-(3-dimethylamino-propyl)carbodiimide
ETD: Everhart Thornley Detector
EthD-1: Ethidium homodimer-1
FBS: Fetal bovine serum
GM: Growth medium
HA: Hyaluronic acid
MHC: Skeletal myosin heavy chain
MyoG: Myogenin
PEI: Polyethyleneimine
PI: Propidium iodide
PLL: Poly-l-lysine
SC: Satellite cell
