# Supplementary material for: Quiescence of human muscle stem cells is favored by culture on natural biopolymeric films
Source: Stem Cell Res Ther. 2017 May 2;8:104. doi: 10.1186/s13287-017-0556-8 (PMC5414338; doi:10.1186/s13287-017-0556-8)
Supplement: Additional file 1: — Figure S1. SCs are alive after 21 days of culture. Calcein/EthD (Live/dead) assay on SCs during 21 days on EDC10 films without any change/refreshing ﻿of ﻿GM. Scale bar: 100 µm. Figure S2. SCs secrete more Collagen I on plastic than on EDC10 films. Immunostaining of fibronectin and collagen I in SCs cultured 21 days on EDC10 films, 3 days on EDC70 fil﻿ms and plastic. Actin was stained by rhodamine-phalloidin. Scale bar: 200 µm. Figure S3. Images of the cells corresponding to Figure 5, for DAPI and Pax7 staining. [file 13287_2017_556_MOESM1_ESM.docx]

**Monge et al, Supporting information**

**FIGURE S1.** **SCs are alive after 21 days of culture**. Calcein/EthD (Live/dead) assay on SCs during 21 days on EDC10 films without any change/refreshing of GM. Scale bar is 100 µm.

**FIGURE S2**. **SCs secrete more Collagen I on plastic than on EDC10 films**. Immunostaining of fibronectin and collagen I in SCs cultured 21 days on EDC10 films, 3 days on EDC70 films and plastic. Actin was stained by rhodamine-phalloidin. Scale bar is 200 µm.

**FIGURE S3.** Images of the cells corresponding to Figure 5, for DAPI and *Pax7* staining. (E) Representative images of actin staining.
